# Supplementary material for: Obesity dysregulates feeding-evoked response dynamics in hypothalamic satiety neurons
Source: Sci Rep. 2025 Oct 14;15:35837. doi: 10.1038/s41598-025-19800-2 (PMC12521569; doi:10.1038/s41598-025-19800-2)
Supplement: Supplementary file 1 — Supplementary Material 1. [file 41598_2025_19800_MOESM1_ESM.pdf]

SUPPLEMENTARY FIGURE 1

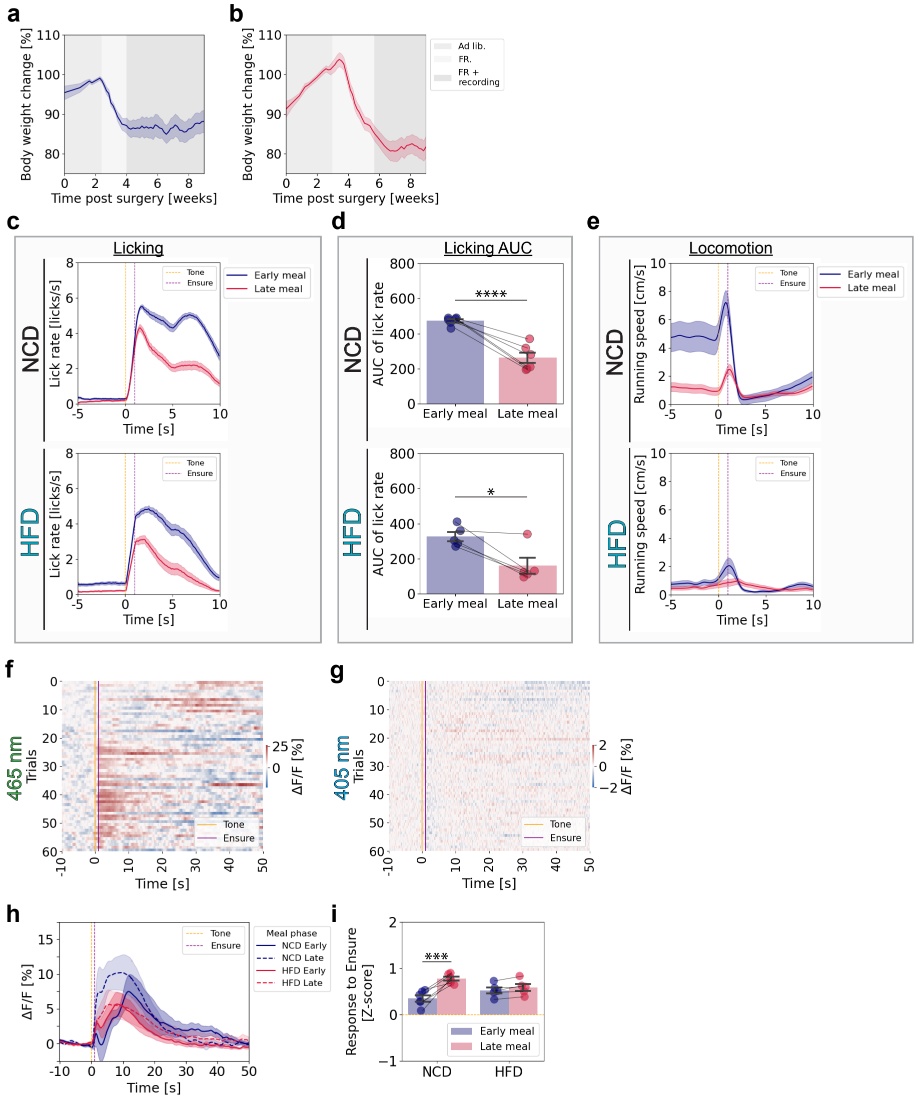

Supplementary Figure 1. Validation of GCaMP6s photometry signals and behavioral dynamics during food restriction in NCD- and HFD-fed mice

a, b, Post-surgical bodyweight dynamics during ad libitum feeding (light gray), food restriction (FR, no shading), and continued food restriction maintenance during the photometry recording (FR + recording, dark shading) of NCD-fed (**a**) and HFD-fed (**b**) mice.

c, Mean lick rate during early and late meal phases for NCD-fed (top panel) and HFD-fed (bottom panel) mice.

d, Area under the curve (AUC) quantification of post-cue lick-rate, normalized to baseline pre-tone lick rate, for early and late meal phases of individual NCD-fed (top panel) and HFD-fed (bottom panel) mice. unpaired, two-tailed Student t-test.

e, Mean running during the early and late meal phase of NCD-fed (top panel) and HFD-fed (bottom panel) mice.

f, g, Representative heatmap of normalized ΔF/F GCaMP6s fluorescent responses detected using a 465 nm LED (**f**) and a 405 nm LED, reflecting movement artifacts and auto-fluorescence (**g**). Responses in each trial are normalized to the 10 s baseline before the cue onset. Note the range in **i** is smaller than in **a**.

h, Early and late meal responses to Ensure for NCD-fed and HFD-fed animals, expressed as ΔF/F.

i, Z-scored PVH^{MC4R} neuronal responses of each animal during early and late meal phases for all recordings in NCD-fed and HFD-fed mice, averaged from 1 – 20 s post-cue presentation. NCD: 6 mice; HFD: 5 mice.

i, Two-way Anova with Tukey post hoc test, for Early vs. Late meal within each condition and early phases between conditions.

a – e, h – i, Data are represented as the mean ± s.e.m either as error bars (**d, i**) or as a shaded area (**a – c, e, h**).

a, b, k, * - P <0.05, *** - P <0.001, **** - P <0.0001.

SUPPLEMENTARY FIGURE 2

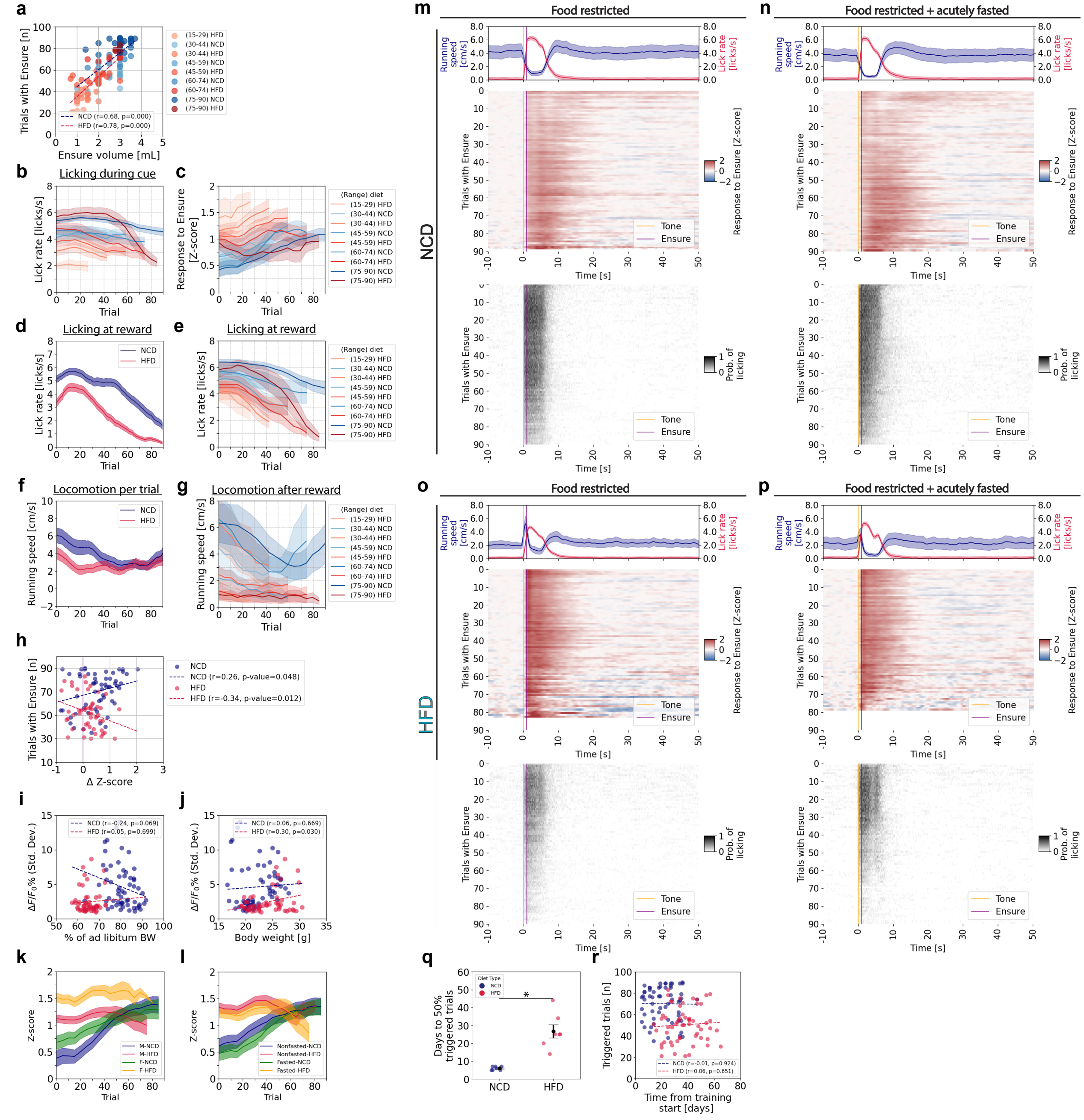

Supplementary Figure 2. Diet- and fasting-dependent modulation of PVH^{MC4R} neuronal activity, licking behavior, and trial engagement in NCD- and HFD-fed mice

a, Meal length vs. consumed Ensure volume grouped by the number of triggered trials (ranges: 15–29, 30–44, 45–59, 60–74, 75–90) in NCD-fed (blue shade) and HFD-fed (red shade) mice. Dashed lines indicate linear fits for NCD and HFD groups.

b, Lick rate during the 1 s cue window grouped by the number of triggered trials (ranges: 15–29, 30–44, 45–59, 60–74, 75–90) in NCD-fed (blue shade) and HFD-fed (red shade) mice.

c, Single trial mean neuronal responses 1-9 s post cue (peri-licking) grouped by the number of triggered trials (ranges: 15–29, 30–44, 45–59, 60–74, 75–90) in NCD-fed (blue shade) and HFD-fed (red shade) mice.

d, Single trial mean licking rate during the reward period (1 – 9 s post-cue) across 90 recorded trials from NCD-fed and HFD-fed groups (minimum 30 successfully triggered trials).

e, Single trial mean licking rate during the reward (1 – 9 s post cue) across 90 recorded trials grouped by the number of triggered trials (ranges: 15–29, 30–44, 45–59, 60–74, 75–90) for NCD-fed (blue shading) and HFD-fed (red shading) mice.

f, Single trial mean running speed across 90 recorded trials from NCD-fed and HFD-fed mice (minimum 30 successfully triggered trials).

g, Single trial mean running speed during the pre-cue period across 90 recorded trials, grouped by the number of triggered trials (ranges: 15–29, 30–44, 45–59, 60–74, 75–90) for NCD-fed (blue shade) and HFD-fed (red shade) mice.

h, Number of triggered trials out of the 90 possible trials recording session versus ΔZ-score between computed by subtracting the mean early meal response from the mean late meal response. The dashed line represents the linear relationship between meal size (triggered trials with Ensure) and the ΔZ-score (i.e. the change in response to Ensure from early to late in the meal).

i, j, Correlation analysis of fluorescence variation quantified as the standard deviation of $\Delta F/F_0$ relative to the animal's maximum body weight (BW) during ad libitum feeding (% of ad lib BW) (**i**), and the absolute body weight on the day of the recording (**j**), separated by NCD (red) and HFD (blue) diet.

k, Single trial mean peri-licking PVH^{MC4R} responses (1 – 9 s post-cue) across 90 recorded trials from female (F) and male (M) NCD-fed and HFD-fed groups. Only recordings with at least 30 successfully triggered trials were included. M-NCD: 26 recordings / 3 mice; M-HFD: 31 recordings / 4 mice; F-NCD: 33 recordings / 4 mice; F-HFD: 23 recordings / 3 mice.

l, Single-trial mean peri-licking PVH^{MC4R} responses (1 – 9 s post-cue) across 90 recorded trials for food-restricted (nonfasted) and food-restricted plus acute fasting (fasted) NCD-fed and HFD-fed groups. Only recordings with at least 30 successfully triggered trials were included.

m – p, Heatmaps summarizing GCaMP6s photometry signals (top panel) and licking behavior (bottom panel) from PVH^{MC4R} neurons in food-restricted-only sessions (**m, o**) and in food-restricted plus acutely-fasted sessions (**n, p**) for NCD-fed (**m, n**) and HFD-fed (**o, p**) animals. Mean running speed and licking rates across all triggered trials are shown above the heatmaps.

Only recordings with a minimum of 30 successfully triggered trials were included. Trial structure: 10 s baseline before cue (tone) onset (t = 0 s), followed by Ensure delivery at t = 1 s.

l – p, Nonfasted NCD: 31 recordings / 7 mice (**l, m**); Food restricted plus acutely fasted NCD: 28 recordings / 7 mice (**l, n**); Nonfasted HFD: 31 recordings / 7 mice (**l, o**); Food restricted plus acutely fasted HFD: 23 recordings / 7 mice (**l, p**).

e – m, Data are represented as the mean ± s.e.m. * - P <0.05, *** - P <0.001, **** - P <0.0001.

c, e, g, NCD: 59 recordings / 7 mice; HFD: 54 recordings / 7 mice.

a, b, d, f, Recordings for each meal size range: NCD (15–29): 0; HFD (15–29): 6; NCD (30–44): 6; HFD (30–44): 13; NCD (45–59): 10; HFD (45–59): 23; NCD (60–74): 13; HFD (60–74): 7; NCD (75–90): 28; HFD (75–90): 4.

SUPPLEMENTARY FIGURE 3

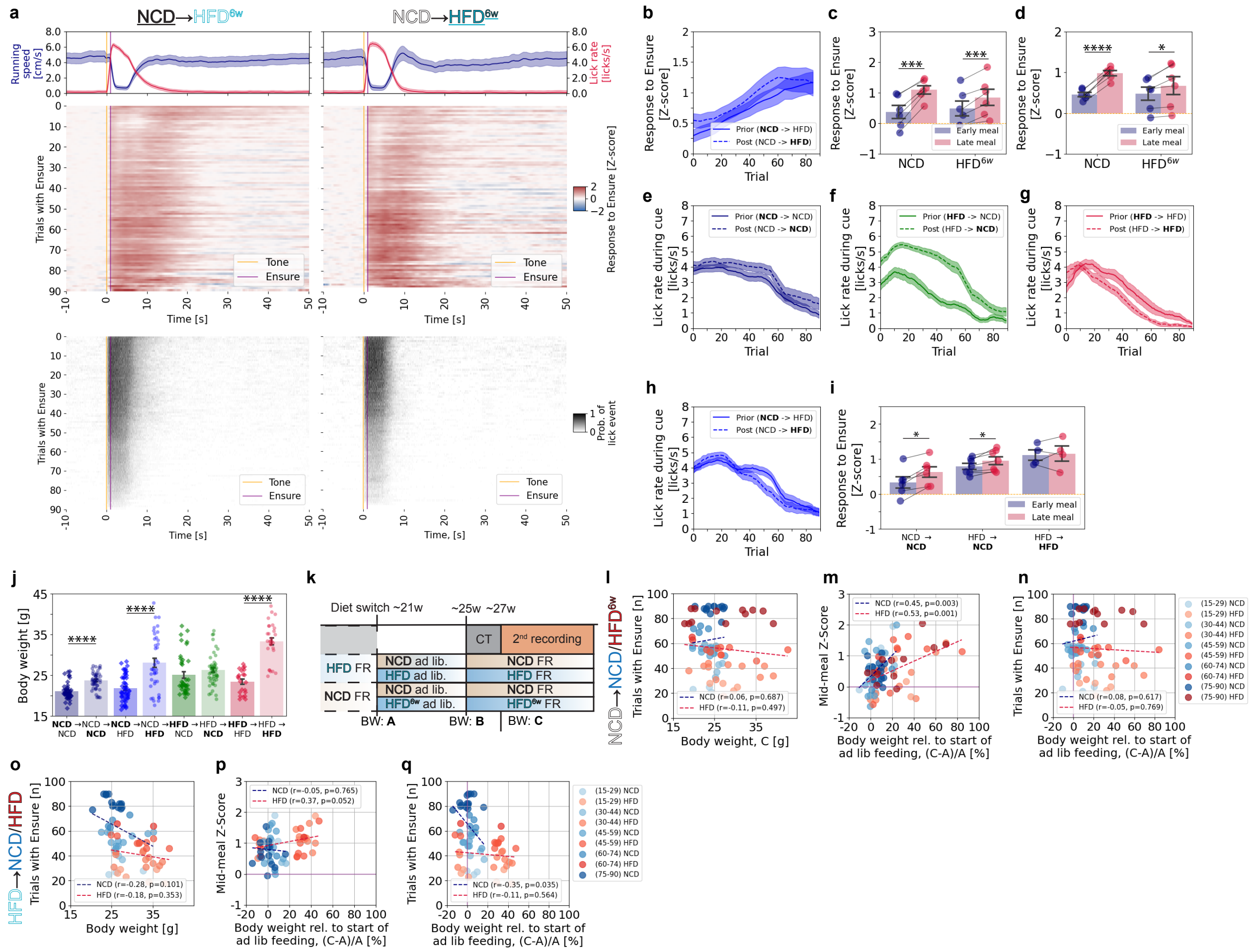

Supplementary Figure 3. Impact of diet switch on cue-evoked licking behavior and PVH^{MC4R} dynamics during early and late meal phases

a, Mean heatmaps summarizing GCaMP6s photometry signals (middle panel) and licking events (bottom panel) in the same NCD→HFD animals during the first phase of NCD diet (left, <u>NCD→</u>HFD) and second phase of <u>HFD</u> diet (right, NCD→<u>HFD</u>^{6w}). Top panel: mean running speed and licking rate across all triggered trials. Only recordings with a minimum of 30 successfully triggered trials were included in the averaged heatmap analyses, while untriggered trials were excluded from the calculation of average photometry and behavior signals.

b, Single-trial mean PVH^{MC4R} neuron responses during food consumption (1 – 9 s post cue) (**b**) across 90 trials across triggered trials from all recordings in which at least 30/90 trials were triggered in the NCD→HFD group.

c, d, Mean Z-scored PVH^{MC4R} neuronal responses (1 – 9 s post cue) (**c**) and (1 – 19 s post cue) (**d**) during early meal (first 15 trials) and late meal (last 15 trials) phases of the meal, for individual

mice (averaged across sessions per mouse) for NCD mice switched to HFD for 6 weeks (HFD^{6w}) or maintained on NCD. Two-tailed paired Student's t-test

e – h, Single-trial mean lick rate during cue presentation (0 – 1 s post-cue onset) across 90 trials averaged across recordings with at least 30 successfully triggered trials in the NCD→NCD (**e**), HFD→NCD (**f**), HFD→HFD (**g**), NCD→HFD^{6w} (**h**) groups, comparing pre-diet-switch (solid line) and post-diet-switch (dashed line) conditions.

i, Mean Z-scored PVH^{MC4R} neuronal responses during early (first 15 trials) and late meal (last 15 trials) phases of the meal, for individual mice (averaged across sessions per mouse) across the following conditions post-diet switch: NCD→NCD, HFD→NCD, HFD→HFD. Paired, two-tailed t-test between early-meal and late-meal trials.

j, Body weights during the individual photometry recording sessions, categorized by groups of NCD→NCD (navy), NCD→HFD^{6w} (blue), HFD→NCD (green), and HFD→HFD (red) groups, comparing pre-diet-switch (diamonds) and post-diet-switch (circles) conditions. Linear mixed model

a – h, j Recording and animal numbers during exposure to the first diet: **NCD**→NCD: 44 recordings / 6 mice; **NCD**→HFD^{6w}: 47 recordings / 6 mice; **HFD**→NCD: 52 recordings / 7 mice; **HFD**→HFD: 35 recordings / 4 mice. Recording and animal numbers during exposure to the second diet: NCD→**NCD**: 43 recordings / 6 mice; NCD→**HFD^{6w}**: 36 recordings / 6 mice; HFD→**NCD**: 42 recordings / 7 mice; HFD→**HFD**: 23 recordings / 4 mice.

k, Experimental paradigm for diet-switch experiments with further 6-week ad libitum exposure of either HFD or NCD after the initial recordings, where concluded, indicating the relevant body weight measurement taken before the ad libitum second diet exposure (A), maximal body weight reached after the ad libitum exposure period (B), and absolute body weight during the recording period (C).

l, Correlation between meal size (number of triggered trials, i.e. trials with Ensure) and absolute body weight in NCD–>NCD and NCD–>HFD^{6w} animals during the second feeding phase.

m, n, Correlation of mid-meal neuronal responses (averaged across the 15th to the 29th triggered trial) with meal size (number of triggered trials, i. e. trials with Ensure) (**m**) and with body weight on the recording day (C, in Supp. Fig. 3k) relative to the start of the ad libitum feeding (A, in Supp. Fig. 3k) (**n**).

o, Correlation between meal size (number of triggered trials, i.e. trials with Ensure) and absolute body weight in HFD –> NCD and HFD –> HFD animals during the second feeding phase

p, q, Correlation of mid-meal neuronal responses and meal size (number of triggered trials, i. e. trials with Ensure) (**p**) and for individual recordings with body weight (see C, in Supp. Fig. 3k) relative to the start of the ad libitum feeding (A, in Supp. Fig. 3k) (**q**).

l – q, values were averaged across the 15th to the 29th triggered trials for individual recordings, with the total number of trials in that session represented by light-to-dark shading (blue: NCD; red: HFD). Dashed lines indicate linear fits for the second diet phase, i.e. NCD and HFD groups.

a – j, Data are represented as the mean ± s.e.m as shaded line or as error bars across sessions (**b, e – h, j**) or across mice (**c, d, i**).

c, d, i, j, * - $P < 0.05$, ** - $P < 0.01$, *** - $P < 0.001$, **** - $P < 0.0001$.
